# Cytosolic β-glucosidase inhibition and renal blood flow suppression are leading causes for the enhanced systemic exposure of salidroside in hypoxic rats

**DOI:** 10.1039/c7ra13295f

**Published:** 2018-02-23

**Authors:** Te Qi, Bei-kang Ge, Liang Zhao, Yi Ma, Xiao-rong Li, Ping-xiang Xu, Ming Xue

**Affiliations:** Department of Pharmacology, Beijing Laboratory for Biomedical Detection Technology and Instrument, School of Basic Medical Sciences, Capital Medical University Beijing China xuem@ccmu.edu.cn +86 10 8391 1520 +86 10 8391 1520

## Abstract

The promising benefits of salidroside (SAL) in alleviating high altitude sickness boost investigations on its pharmacokinetics and biological activity. However, the transportation and disposition process of SAL under hypoxic conditions has never been explored. The current study was proposed to investigate the pharmacokinetics of SAL in hypoxic rats and to explore the underlying mechanisms for the distinct metabolic fate of SAL under hypoxia. Pharmacokinetic studies on SAL was conducted in both hypoxic and normoxic rats. The transport properties of SAL were investigated on both hypoxic and normoxic Caco-2 monolayer models. Enzymes involved in SAL metabolism were identified and the effects of hypoxia on these enzymes were assessed by real-time PCR, western blotting analyses, and rat liver homogenate incubation. The renal clearance (CL_r_) of SAL, effective renal plasma flow (ERPF) and glomerular filtration rate (GFR) in both hypoxic and normoxic rats were also determined for renal function assessment. It was found that the systemic exposure of SAL in hypoxic rats was remarkably higher than that in normoxic rats. The barrier function of Caco-2 monolayer was weakened under hypoxia due to the impaired brush border microvilli and decreased expression of tight junction protein. Hepatic metabolism of SAL in hypoxic rats was attenuated due to the reduced activity of cytosolic β-glucosidase (CBG). Moreover, CL_r_ of SAL was reduced in hypoxic rats due to the suppressed ERPF. Our findings suggest the potential need for dose-adjustment of SAL or its structural analogs under hypoxic conditions.

## Abstract 2

CBG inhibition and renal blood flow suppression are leading causes for the enhanced systemic exposure of SAL in hypoxic rats.

## Graphical abstract

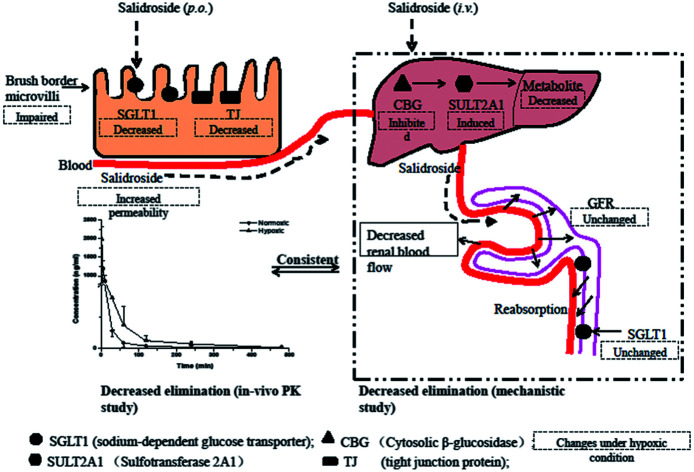

## 1. Introduction

**Fig. 1:**
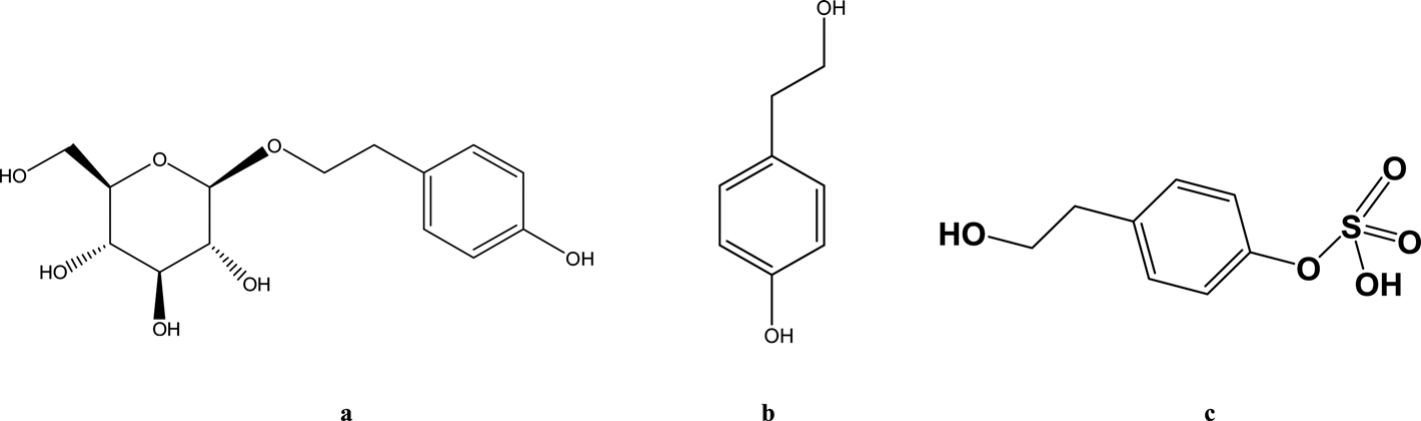
The chemical structures of salidroside (a), *p*-tyrosol (b) and *p*-tyrosol sulfate (c).

Hypoxia triggers a sequence of physiological and pathological events. The common causes of hypoxia include diseases, high altitude and aerospace traveling. Accumulating studies demonstrate that the pharmacokinetics of some drugs such as ibuprofen and sulfamethoxazole are altered when animals or humans are subjected to hypoxic conditions.^1^*Rhodiola rosea* L. is widely used in Asia and Eastern Europe for anti-hypoxia and preventing high altitude sickness.^2–4^ Salidroside (SAL) (Fig. 1a) is reported as a major and crucial constituent accounting for the anti-hypoxic activity of Rhodiola. Our preliminary studies indicate an enhanced systemic exposure and reduced *in vivo* clearance of SAL in hypoxic rats when compared with that in normoxic rats. Such findings entail subsequent mechanistic investigations on the absorption and disposition processes of SAL under hypoxia conditions.

Considering SAL belongs to the phenolic glycoside family, some researchers suggest that glucose transporters may be involved in the absorption of SAL.^5–8^ He *et al.* suggested that sodium-dependent glucose transporter (SGLT1) plays an important role in the absorption of SAL in rats.^9^ Findings from literature and our preliminary studies both suggested that after oral administration of SAL to rats, more than 50% of the parent compound undergoes deglycosylation and sulfation. The major metabolite (Fig. 1c) in systemic circulation turns out to be the sulfate form of SAL aglycone (*p*-tyrosol).^10,11^ There are indeed speculations suggesting that hypoxia alters pharmacokinetics of some drug by suppressing the cytochrome P450 (CYP) enzymes, but the effect of hypoxia on SGLT1, glycosidase and sulfotransferase (SULT) has never been explored.^12^ Since about 20% of administered SAL is excreted from urine in the form of parent compound, whether hypoxia alters the renal function of rats also deserve further investigation.

In the current study, we firstly described the differences in pharmacokinetic profiles of SAL between the normoxic and hypoxic rats. Mechanistic studies were subsequently conducted to explain the distinct pharmacokinetics of SAL under hypoxic condition. Specifically, the absorption process of SAL under hypoxia was studied on basis of the hypoxic Caco-2 monolayer model. Transporter and enzymes involved in the *in vivo* absorption and disposition of SAL were identified with the aid of specific inhibitors. The expression levels and metabolic activities of these enzymes in normoxic and hypoxic rats were then measured and compared. In addition, the renal clearance (CL_r_) of SAL, the effective renal plasma flow (ERPF) and the glomerular filtration rate (GFR) of hypoxic and normoxic rats were determined for renal function assessment.

## 2. Materials and methods

### 2.1. Materials

SAL (batch no. 43866) and *p*-tyrosol (batch no. 90312) with purity over 98% were obtained from Sigma-Aldrich Company (St. Louis, Mo, USA). Paracetamol (purity ≥ 98%), served as the internal standard (IS), were supplied by Shanghai Yuanye Bio-Technology Co. Ltd (Shanghai, China). Phlorizin, taurocholic acid and conduritol B epoxide were purchased from Med Chem Express (Shanghai, China). Primary and secondary antibodies for SULT2A1 and the nuclear pregnane X receptor (PXR) were purchased from Abcam (Cambridge, UK). Antibodies for cytosolic β-glucosidase (CBG) were obtained from Bioss Bio-Technology Co. Ltd (Beijing, China). Antibodies for SGLT1 and zonula occludens-1 (ZO-1) were purchased from Cell Signaling Technology (Beverly, MA, USA). Antibodies for hypoxia inducible factor-1α (HIF-1α) were purchased from Novus Biologicals, Inc., Littleton, CO, USA. Lactate dehydrogenase (LDH) assay kit, citrate synthase (CS) assay kit, and HIF-1α ELISA kit were obtained from Nanjing Jiancheng Bioengineering Institute (Nanjing, China). Methanol and acetonitrile of HPLC grade were purchased from Thermo Fisher Technologies Inc (Waltham, MA, USA). Distilled and deionized water from Wahaha Co. Ltd (Hangzhou, China) was used for the preparation of all solutions. Other reagents were all of analytical grade.

### 2.2. Animals

Healthy male Sprague-Dawley (SD) rat (6–8 week-old, 250 ± 20 g), a recognized animal model used for pharmacokinetic study, were supplied by the Animal Center of Capital Medical University (ACCMU, Beijing, China). The rats were housed in a room with controlled illumination (a 12 h light and dark cycle), temperature (22 ± 2 °C) and 23% relative humidity for one week to acclimatize. Up to three rats were housed in individually ventilated cages with at least 2 cm of wood shavings bedding and free access to food pellets and water. After surgery, and during measurements as appropriate, rats were housed in individual cages. All the rat experiments were operated in accordance with the Guide for the Care and Use of Laboratory Animals as adopted and promulgated by the National Health Ministry of China. The procedures of rat experiments had been approved by the Animal Center of Capital Medical University.

### 2.3. Establishment of hypoxic rat model

The hypoxic rat model was established using our previously published method.^13^ Briefly, rats were exposed to hypoxic condition (oxygen content of 9%) for 3 consecutive days to construct the model. Normoxic controls were kept in room air (21% O_2_) for 3 consecutive days. The concentrations of LDH, CS, and HIF-1α in rat plasma were monitored with the aid of commercial kit listed in ‘Material’ section for model validation.

### 2.4. Determination and quantification of SAL in biological matrices

The UPLC-MS/MS system consisted of Agilent 1290 series LC pumps and auto-sampler (Agilent, CA, USA), coupled with an Agilent 6490 triple quadrupole mass spectrometer (Agilent Technologies, CA, USA) equipped with electrospray ionization (ESI). Chromatographic separation was achieved by Waters Acuquity HSS T3 (2.1 mm × 100 mm, 1.8 μm) with a column temperature of 40 °C. The mobile phase was composed by solvent A (0.2% formic acid in water) and solvent B (acetonitrile). Gradient elution was performed according to the following program: the percentage of solvent B started at 5% and linearly increased to 50% in the following 4 min; after maintenance at 50% for 1 min, the percentage of B was returned to 5% in 1 min and equilibrated for another 1 min for the next injection. The flow rate was set as 0.2 ml min^−1^ and the total running time was 7 min. The temperature of the auto-sampler was set at 4 °C. The MS/MS system was operated under negative mode with an optimized condition as follow: ion spray voltage at +5.5 kV; nitrogen as nebulizer gas, auxiliary gas and curtain gas at 30, 60 and 10 psi, respectively; auxiliary gas temperature at 225 °C and interface heater temper at 110 °C. The multiple reaction monitoring (MRM) analyses was conducted by monitoring the precursor ion to production transitions, including *m*/*z* 345.0 → 299.1 for SAL, *m*/*z* 137.1 → 119.0 for *p*-tyrosol, *m*/*z* 217.0 → 137.1 for *p*-tyrosol sulfate and *m*/*z* 150.2 → 106.9 for paracetamol (internal standard, IS). The retention time of SAL, *p*-tyrosol, *p*-tyrosol sulfate, and IS was determined to be 3.7, 4.9, 3.1 and 4.5 min, respectively.

Seven serial concentrations, ranged from 5 to 2500 ng ml^−1^, were used to establish the calibration curves for the quantification of SAL. To prepare the samples for calibration curves, 10 μl of IS working solution (containing 100 μg ml^−1^ paracetamol) and 10 μl of analyte work solution was added to 100 μl of blank rat plasma. Approximately 300 μl methanol was then added to the above plasma sample for protein precipitation. After centrifuging the mixture at 11 300*g* for 10 min, the supernatant was collected followed by being evaporated to dryness under nitrogen at room temperature. The residue was reconstituted with 100 μl of methanol. After being centrifuged at 11 300*g* for 10 min, an aliquot of 5 μl supernatant was injected into the UPLC-MS/MS system for analyses. Under the present chromatographic conditions, no endogenous interference from rat biological matrices was observed at the retention time for the analyte and IS. Calibration curves of SAL in biological matrices including plasma, urine and liver homogenate were linear over 5–2500 ng ml^−1^ concentration ranges with regression coefficients (*r*^2^) above 0.995. The limit of quantification (LOQ) of SAL for the current assay was 5 ng ml^−1^. For all QC samples in rat biological matrices, the % RSD of both intra-day and inter-day precision was below 10.4%, and the accuracy was within the range of 85.4 to 100%, which met the criteria from FDA. The extraction recoveries of SAL at three QC concentrations ranged from 87.2 to 94% and were consistent over the concentration range examined. Moreover, SAL was found to be stable both in the auto-sampler (4 °C) for 24 h and in −80 °C for 30 days. No significant matrix effect was observed for the current one-step UPLC/MS/MS method.

### 2.5. Assessing the pharmacokinetics of SAL in hypoxic rats and normoxic rats

Twenty male SD rats were randomly divided into hypoxic group (HYP) and normoxic group (NOR) with ten rats in each group. Following hypoxic and normoxic model construction, rats were prepared for surgery. The protocol of rodent preparation and jugular vein cannulation has been previously described in detail.^14^ Briefly, rats were anesthetized with an intramuscular dose of ketamine (90 mg kg^−1^) and xylazine (9 mg kg^−1^) followed by cannulation with a polythene tube (0.5 mm i.d., 1 mm, Portex Ltd., Hythe, Kent, England) in the right jugular vein. The rats were allowed to recover overnight and had free access to water and food. SAL was then administered to both rats in HYP group and NOR group by oral and by intravenous to form four subgroups (*n* = 5), including the hypoxic rats with oral administration of SAL group (HYP_PO_), the hypoxic rats with intravenous administration of SAL group (HYP_IV_), the normoxic rats with oral administration of SAL group (NOR_PO_), and the normoxic rats with intravenous administration of SAL group (NOR_IV_). For oral groups, SAL was dissolved in saline and given to rats at 4.44 mg kg^−1^ by gavage. As to intravenous group, SAL was given to rats at 444 μg kg^−1^ by an i.v. bolus injection through the tail-veins. The blood samples (200 μl) were taken from the jugular vein catheter and were collected into the heparinized tubes at the following time points: 0, 5, 10, 30, 60, 120, 240, 360, 480, 720 and 1440 min after oral administration and 0, 2, 5, 10, 30, 60, 120, 240, 360, 480 min after intravenous administration, respectively. An aliquot of 200 μl sterile isotonic saline was given immediately to rats after blood sample collection for compensation of body fluid loss. The animals were sacrificed with pentobarbital sodium salt (200 mg kg^−1^, i.p.) at the end of the experiment. The obtained blood samples were centrifuged at 11 300*g* for 5 min at 4 °C and the plasma samples (supernatant) was collected for UPLC-MS/MS analyses. Protein precipitation was adopted for samples preparation.

### 2.6. Assessing the transport of SAL in Caco-2 monolayers under hypoxia and normoxia

#### 2.6.1. Establishment of hypoxic Caco-2 monolayer cell model

The human colorectal adenocarcinoma cell line, Caco-2 cell, was obtained from Cell Resource Center, Institute of Basic Medical Sciences (CAMS). Caco-2 cells, a recognized cell model used for permeability study, were cultured in minimum essential medium (MEM) containing 10% fetal bovine serum, 1% nonessential amino acids, 100 U ml^−1^ penicillin, and 100 μg ml^−1^ streptomycin, and then were kept in a humidified incubator at 37 °C with 5% CO_2_. To obtain differentiated monolayers, Caco-2 cells were seeded in Transwell® chambers (12 mm, 0.4 μm pore size; Corning, MA, USA) at a density of 1 × 10^5^ cells per wells and cultured for 21 days. TEER (trans-epithelial electrical resistance) was used to monitor the integrity of the monolayer. Monolayer with TEER above 600 Ω cm^2^ was employed in the present study. Caco-2 cells grown in Transwell® at passage 32–45 were used for the experiment. After cultivation for 21 days, normoxic incubations were performed in a tissue culture incubator at 37 °C, 5% CO_2_, while hypoxic incubations were performed in an oxygen-control hypoxic glove box at 37 °C, 1% oxygen content for 24 h.^15^ The enzyme activities of LDH, CS and the protein expression of HIF-1α were monitored for hypoxic model validation. The morphological study on hypoxic Caco-2 monolayer was performed with the aid of transmission electron microscopy (TEM) (JEM-2100F, Japan). The expression level of tight junction proteins (ZO-1) in Caco-2 cell under normoxic and hypoxic conditions was measured by western blotting.

#### 2.6.2. Determination of the apparent permeability coefficient (*P*_app_) of SAL

According to the method published in *Nature Protocols*, the transport test was performed in Hank's balanced salt solution (HBSS) containing 5.56 mM glucose, 10 mM HEPES and 1.26 mM CaCl_2_.^16^ Before experiment, the monolayers were washed twice with HBSS medium and pre-incubated for 30 min. SAL was added to either apical (AP) or basolateral (BL) side for the preparation of donor solution with a final concentration of 10 μM. An aliquot of 100 μl samples were then taken from the receiver side at 0, 30, 60, 90, 120, 150, and 180 min after addition of SAL. Equal volume of the blank HBSS buffer was added back to the chambers after sampling to maintain a constant systemic volume. The collected samples were stored at −20 °C until UPLC-MS/MS analyses. The *P*_app_ was calculated according to the following equation: *P*_app_ = [(d*C*/d*t*) × *V*]/[*A* × *C*_0_], where d*C*/d*t* (mg ml^−1^ s^−1^) represents the change of accumulated SAL concentration overtime in the receiver chamber, *V* (cm^3^) represents the solution volume in the receiver chamber, *A* (cm^2^) represents surface area of the Caco-2 monolayer membrane (1.12 cm^2^), *C*_0_ (mg ml^−1^) represents the loading concentration of SAL in donor chamber.

#### 2.6.3. Identification of the transporters involved in the absorption of SAL

A well-known SGLT1 inhibitor, phlorizin, was used to characterize the potential uptake transporters involved in SAL absorption.^9^ The Caco-2 monolayer was pre-incubated with phlorizin (100 μM) for 30 min before the transport experiment. The *P*_app_ of SAL incubated with phlorizin was also determined as described above.

### 2.7. Assessing the hepatic metabolism of SAL under hypoxia and normoxia

*p*-Tyrosol sulfate was the predominant metabolite identified in the hepatic metabolism of SAL, which accounted for over 50% of the total metabolites in systemic circulation.^10,11^ The formation rate of *p*-tyrosol sulfate could therefore serve as an indicator for the hepatic metabolic rate of SAL. The liver S9 fraction (RLS) derived from 5 normoxic rats and 5 hypoxic rats were prepared respectively according to previously mentioned method.^17^ Briefly, after rat liver perfusion, the whole-liver homogenate was prepared immediately on ice. RLS, consisting of both microsomal and cytosolic fractions, was obtained by centrifugation of above homogenate sample at 9000*g* for 20 min. The animals were sacrificed with pentobarbital sodium salt (200 mg kg^−1^, i.p.) at the end of the experiment. All surgical procedures were performed under anesthesia and in a clean surgical room with sterilized instruments. All efforts were made to minimize the suffering of the animals during the experiments. To evaluate the kinetic process of deglycosylation and sulfation, SAL (0.1–100 μM) or *p*-tyrosol (0.05–50 μM) was pre-incubated respectively with 3.2 mg ml^−1^ RLS in 50 mM Tris–HCl buffer (pH 7.4) containing 8 mM MgCl_2_ and 25 μg ml^−1^ of alamethicin for 5 min. The reaction was initiated by adding 2 mM 3′-phosphoadenosine-5′-phosphosulfate (PAPS). The incubation was carried out at 37 °C and terminated by the addition of an equal volume of ice-cold methanol containing 5 μM IS.^18^ The content of *p*-tyrosol sulfate was determined by the UPLC-MS/MS described above. Exact quantification of *p*-tyrosol sulfate could not be performed due to the lack of authentic standards. Thus, a semi-quantitative approach was adopted, in which the quantities of *p*-tyrosol sulfate was estimated from the calibration curve of its parent compound-SAL.^18,19^ Specifically, after obtaining the peak area ratio of each *p*-tyrosol sulfate to IS, the ratio was fitted into the weighted linear least-squares regression equation of the calibration curve of SAL. Corresponding molar concentrations of *p*-tyrosol sulfate was then estimated. All the experiments were carried out in triplicate.

To screen the potential β-glycosidase and SULT isozymes involved in the hepatic metabolism of SAL, six well-known isoform-selective inhibitors were used including conduritol B epoxide for glucocerebrosidase (50, 100, 500, 1000, 2000 μM), taurocholic acid for CBG (0.5, 1, 10, 100, 1000 μM), quercetin for SULT2A1 (1, 10, 50, 100, 500 μM), nimesulide for SULT 1A1 (1, 10, 50, 100, 500 μM), ibuprofen for SULT 1E1 (1, 10, 50, 100, 500 μM), and kynurenic acid for SULT 1B1 (1, 10, 50, 100, 500 μM).^20–22^ The total volume of the incubation mixture was 200 μl, containing RLS (1 mg ml^−1^), 100 mM potassium phosphate buffer (pH 7.4), different isoform-selective inhibitors, SAL (12.5 μM) or *p*-tyrosol (12.5 μM) and 2 mM of PAPS. Reactions were initiated by the addition of PAPS. Negative control incubation was performed in the absence of the corresponding isoform inhibitors. Incubations were conducted at 37 °C for 60 min and terminated by the addition of 200 μl of ice-cold methanol containing 5 μM IS. The content of *p*-tyrosol sulfate was determined on basis of UPLC-MS/MS method.

### 2.8. Assessing the renal function of hypoxic and normoxic rats

#### 2.8.1. Determination of the CL_r_ of SAL and *p*-aminohippuric acid (PAH) in hypoxic and normoxic rats

The animal surgery was conducted as described in literature.^23,24^ Briefly, the cannulae were inserted into rat right jugular veins, left femoral veins, and bladders after an i.m. injection of 90 mg kg^−1^ ketamine and 9 mg kg^−1^ xylazine. All surgical procedures were performed under anaesthesia and in a clean surgical room with sterilized instruments. All efforts were made to minimize the suffering of the mice during the experiments. Urine from the bladder was collected through the catheter into pre-weighed micro-centrifuge tubes and the volume of urine collected during each 30 min period was calculated by weight. To equilibrate and establish a steady rate of urine excretion, rats were continuously infused with warm saline solution containing mannitol (5%, w/v) for 1 h at a rate of 16 μl min^−1^ through the femoral veins (*n* = 5 each group) using a Sage Instruments Syringe Pump (Cambridge, MA, USA). During the experiment, the rat was placed on a heating pad to maintain body temperature. After equilibration, SAL (444 μg kg^−1^) or PAH (30 mg kg^−1^) was administered by an i.v. bolus injection to rats through the femoral veins. Blood samples (200 μl each) were drawn from the jugular vein at 0, 2, 5, 10, 30, 60, 120, 240, 360, 480 min after dosing. After each blood sampling, an aliquot of 200 μl saline was infused into the jugular to replace the volume of blood taken. The animals were sacrificed with pentobarbital sodium salt (200 mg kg^−1^, i.p.) at the end of the experiment. The concentration of SAL or PAH in rat plasma and urine samples was measured respectively by the validated UPLC-MS/MS method mentioned above or by an UPLC-MS/MS published previously.^25^

#### 2.8.2. Determination of the serum concentration of creatinine in hypoxic and normoxic rats

Accurate measurement of GFR is complicated and costly. Popper and Mandel proposed the use of serum creatinine in previously published report, which remains the most widely used marker for GFR estimation in spite of its shortcoming.^26^ The serum concentration of creatinine in hypoxic and normoxic rats was measured by the commercial Creatinine Assay Kit (Abcam, Shanghai, China).^27^

#### 2.8.3. Determination of the protein binding of SAL in hypoxic and normoxic rats

Protein binding determinations of SAL in rat plasma were performed using ultrafiltration method. SAL of different concentration, including 100, 500, and 1000 μg ml^−1^, was incubated with plasma sample from hypoxic and normoxic rats (*n* = 5), respectively. After incubation at 37 °C for 30 min, an aliquot of 500 μl plasma was added into the upper part of the centrifugal filter device (Amicon®, cutoff value: 10 kDa). The centrifuge was then conducted in a rate of 11 300*g* for 30 min at room temperature. SAL in the original sample and ultrafiltrate were determined by UPLC-MS/MS method.

### 2.9. Assessing expressions of SGLT1, ZO-1, CBG, SULT2A1 and PXR under hypoxia and normoxia

#### 2.9.1. Quantitative real-time RT-PCR (qRT-PCR) assay

Both hypoxic and normoxic rats were sacrificed by pentobarbital sodium (200 mg kg^−1^, i.p.). The whole liver derived from five normoxic and five hypoxic rats were collected and homogenized, respectively. Total RNA was extracted with the Qiagen RNA extraction kit (Valencia, CA, USA). The mRNA expression levels of CBG, SULT2A1 and PXR were determined through qRT-PCR following MIQE guidelines. GAPDH was used as housekeeping gene. The PCR primers were designed as follows: GAPDH, 5′-catgaccacagtccatgcca-3′ (forward), and 5′-cagggatgatgttctgggct-3′ (reverse); CBG, 5′-gagagaactgggcttccttca-3′ (forward), and 5′-cacccacagtagactttgaatagtt-3′ (reverse); PXR, 5′-tggccgatgtgtcaacctac-3′ (forward), and 5′-ttctggaagccgccattagg-3′ (reverse); SULT2A1, 5′-tatctgggatcgctcaccct-3′ (forward), and 5′-caccttggccttggaactga-3′ (reverse). The fold change of target gene expression level was determined using the equation as follow: fold change = 2^−Δ(Δ*C*_t_)^, where Δ*C*_t_ = *C*_t(target)_ − *C*_t(GAPDH)_ and Δ(Δ*C*_t_) = Δ*C*_t(hypoxia)_ − Δ*C*_t(normoxia)_

#### 2.9.2. Western blotting analyses

Both hypoxic and normoxic rats (*n* = 5 for each group) were sacrificed by pentobarbital sodium (200 mg kg^−1^, i.p.). The collected rat livers and kidneys were perfused with ice-cold PBS followed by homogenization. Caco-2 cells and rat homogenized tissues were lysed in RIPA buffer containing protease and phosphatase inhibitors (Roche, Mannheim, Germany). Approximately 60 μg of prepared protein samples were used for western blotting analyses. After being resolved on sodium dodecyl sulfate (SDS)-PAGE, the target protein was transferred onto PVDF membranes. The membrane was then blocked in 5% defatted milk followed by incubation with the primary and secondary antibodies. Thereafter, the membrane was immersed in the enhanced chemiluminescence solution (Millipore, Billerica, MA, USA) for 60 s. Digital chemiluminescence images were captured and analyzed by FluorChem Q Imaging System (Alpha Innotech Corporation, Santa Clara, CA).

### 2.10. Data and statistical analyses

GraphPad Prism® was employed to analyze enzyme kinetic data. The Michaelis–Menten equation was used to fit the result of a single experiment with three replicates into non-linear regression. The metabolism of SAL in RLS were reflected by the amount of *p*-tyrosol sulfate formed per min per mg protein (nmol min^−1^·mg^−1^). If the Eadie–Hofstee plot is linear, formation velocity (*V*) of *p*-tyrosol sulfate from SAL or from *p*-tyrosol at respective substrate concentrations ([S]) is fit to the typical form of the Michaelis–Menten equation, which is expressed as *V* = (*V*_max_ × [S])/(*K*_m_ + [S]), where *K*_m_ is the Michaelis constant and *V*_max_ is the maximum formation rate. The intrinsic clearance (Cl_int_), representing *in vitro* intrinsic clearance value of SAL, is calculated as a ratio of *V*_max_ to *K*_m_.^28^

The plasma concentrations *vs.* time profiles of SAL were plotted and analyzed with the aid of WinNonlin (Pharsight Corporation, Mountain View, CA, USA, version 2.1). Pharmacokinetic parameters including the area under the curve (AUC) and elimination half-life (*t*_1/2_) were calculated by non-compartmental model. The peak plasma concentration (*C*_max_) and the time for reaching *C*_max_ (*T*_max_) were obtained directly from the experimental data. The total body clearance (CL_t_) of SAL after intravenous administration is calculated as follows: CL_t_ = dose/AUC. The renal clearance (CL_r_) of SAL after intravenous administration is calculated as follows: CL_r_ = total amount excreted in urine/AUC.

All the data were expressed as mean ± SD. Image processing and image analyses were performed using Image J software, version 1.43 for Windows. Statistical analyses were performed on GraphPad Prism 4.02 for Windows (GraphPad Software Inc., La Jolla, CA, USA). Statistical differences between hypoxic and normoxic groups were calculated using Student's *t*-test. *P* value less than 0.05 was considered to be of statistical significance. For all data, *n* corresponds to independent values. Sample size was determined through power analyses using preliminary data obtained in our laboratory with the following assumptions: ‘*α*’ of 0.05 (two-tailed) and power of 90%. The investigator responsible for data analyses was blinded to which samples/animals represents control and treatment groups. For clarity, most values presented in the figures were normalized to control/baseline values to minimize unwanted sources of variation. The data and statistical analyses comply with the recommendations on experimental design and analyses in pharmacology.

## 3. Results

### 3.1. Enhanced systemic exposure and reduced clearance (CL_t_) of SAL in hypoxic rats

**Fig. 2:**
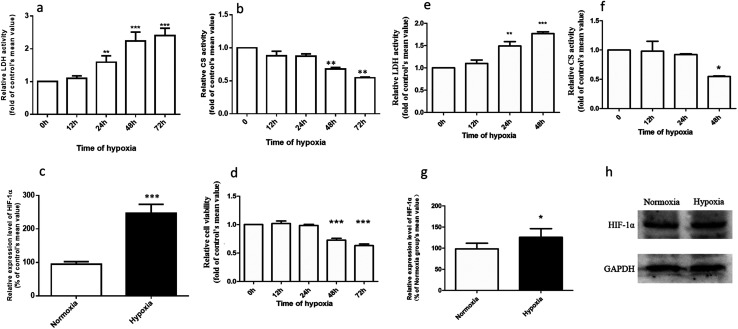
Validation of hypoxic rat model (a–c) and hypoxic Caco-2 monolayer model (d–f), *n* = 5. Assessment of hypoxia-related parameters after exposing rats to hypoxia for 0, 12, 24, 48, and 72 hours. The parameters included the activities of LDH (a), the activity of CS (b) and the expression of HIF-1α (c) in rat plasma. Assessment of hypoxia-related parameters after exposing Caco-2 cells to hypoxia for 0, 12, 24, and 48 hours. The parameters included cell viability (d), the activity of LDH (e), the activity of CS (f) and the expression of HIF-1α (g and h). All data were reported as means ± SD. *p < 0.05, **p < 0.01, ***p < 0.001 *versus* normoxic (0 h) group.

**Fig. 3:**
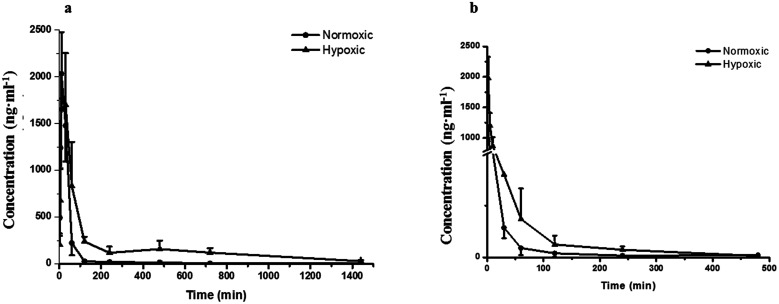
Pharmacokinetic profiles of salidroside in normoxic and hypoxic rats after single oral (a) and intravenous (b) administration at a dose of 4.44 mg kg^−1^ and 444 μg kg^−1^, respectively. *n* = 5. All data were reported as means ± SD.

Fig. 2a–d demonstrates that the hypoxic rat model was successfully established, with an increased LDH activity, decreased CS activity, and up-regulated HIF-1α expression in rat plasma. The plasma concentration *versus* time profiles of SAL in HYP_PO_ group, NOR_PO_ group, HYP_IV_ group and NOR_IV_ group are shown in Fig. 3. The main pharmacokinetic parameters of SAL in these groups calculated by WinNonlin are presented and compared in Table 1. It is noted that compared with that in NOR_PO_ group, AUC_(0→24h)_ of SAL in HYP_PO_ group was significantly higher, which represented an enhanced systemic exposure of this compound in hypoxic rats. The MRT_(0→24h)_ and *t*_1/2_ of SAL in HYP_PO_ group were also significantly prolonged, indicating a slower *in vivo* elimination compared with that in NOR_PO_ group. Similarly, AUC_(0→8h)_ of SAL in HYP_IV_ group was significantly higher when compared with that in NOR_IV_ group. The CL_t_ of SAL was remarkably reduced in HYP_IV_ group (474.0 ± 65.1 ml h^−1^ kg^−1^) in contrast with that in NOR_IV_ group (1140.0 ± 144.3 ml h^−1^ kg^−1^).

**Table 1:** Pharmacokinetic parameters of SAL in normoxic and hypoxic rats (*n* = 5) after oral or intravenous administration^a^

Parameter (unit)	SAL			
	NOR_PO_	HYP_PO_	NOR_IV_	HYP_IV_
*C*_max_ (ng ml^−1^)	2065.3 ± 263.6	1835.4 ± 690.5	N.A.	N.A.
*T*_max_ (min)	36.0 ± 13.4	48.0 ± 16.4	N.A.	N.A.
AUC_(0→t)_ (ng ml^−1^ min)	164 742 ± 11 984	342 569 ± 101 080*	23 144 ± 6060	55 486 ± 22 596*
AUC_(0→∞)_ (ng ml^−1^ min)	165 437 ± 12 176	358 178 ± 102 876*	23 338 ± 6010	56 406 ± 22 317
MRT_(0→t)_ (min)	78.4 ± 19.2	288.0 ± 54.6***	48.3 ± 10.3	70.9 ± 7.9*
*t*_1/2_ (min)	118.4 ± 12.4	365.3 ± 40.0*	67.2 ± 11.8	97.5 ± 12.5*
CL_t_ (ml h^−1^ kg^−1^)	N.A.	N.A.	1140.0 ± 144.3	474.0 ± 65.1**

a Data presented as mean ± SD. *C*_max_: maximum plasma concentration; *T*_max_: time to reach the maximum plasma concentration; AUC_0→∞_: area under the plasma concentration–time curve from 0 h to infinity; *t*_1/2_: elimination half-life; CL_t_: *in vivo* clearance; MRT: mean retention time. N.A.: not available. *p < 0.05, **p < 0.01, ***p < 0.001: statistical significance when compared between HYP_PO_ and NOR_PO_, HYP_IV_ and NOR_IV_.

### 3.2. Dysfunction of hypoxic Caco-2 monolayer resulted in an increased absorption of SAL

#### 3.2.1. Establishment of hypoxic Caco-2 monolayer model

According to our preliminary trials, hypoxic Caco-2 monolayer model was established by exposing Caco-2 monolayers to an environment with 1% O_2_ concentration for 24 h. The enzyme activities of LDH and CS, the protein expression of HIF-1α, and the Caco-2 cell viability were monitored for hypoxic model validation. Fig. 2e–h demonstrates that the hypoxic model was successfully constructed, with an increased LDH activity, up-regulated HIF-1α expression and remained Caco-2 cell viability.

#### 3.2.2. Abnormal morphology and down-regulated ZO-1 expression of Caco-2 monolayer under hypoxia

**Fig. 4:**
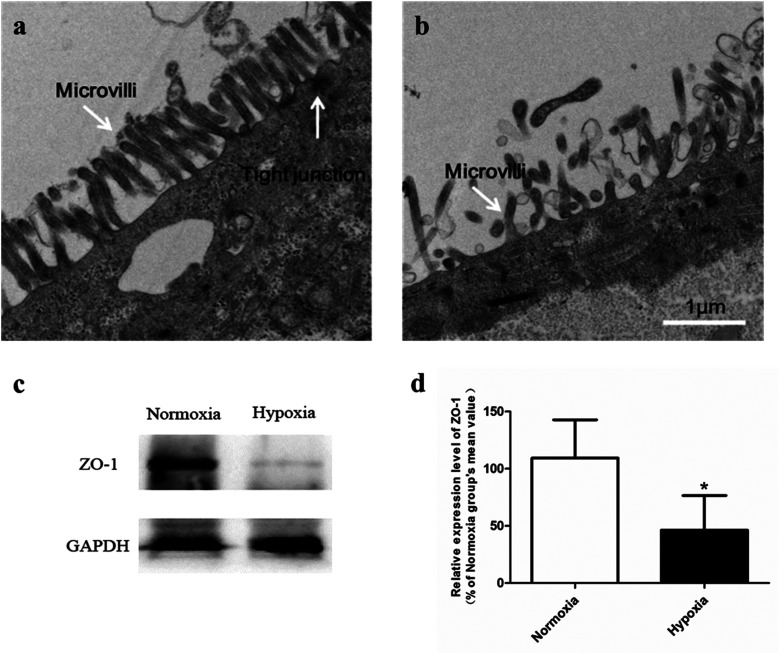
Transmission electron micrographs (TEM) of normoxic Caco-2 cells (a) and hypoxic Caco-2 cells (b). Bar, 1 μm. The expression level of tight junction protein (ZO-1) in normoxic and hypoxic Caco-2 cells (c and d), *n* = 5. All data were reported as means ± SD. *p < 0.05, **p < 0.01, ***p < 0.001 *versus* normoxia group.

Ultrastructure of Caco-2 cell was shown by transmission electron microcopy (Fig. 4a and b), from which the abnormal morphology of hypoxic model was observed. Compared with normoxic Caco-2 cell in which the brush border microvilli was intense, uniform and well developed, the microvilli in hypoxic Caco-2 cells was poor and collapsed. The relative surface area of Caco-2 monolayers was significantly reduced due to the impaired brush border microvilli. The amount of transporters, such as SGLT1,^29^ located in the microvilli may therefore decrease. In addition, the presence of electron-dense material in the space between cells near the brush border reflects the tight junction protein (ZO-1). In normoxic Caco-2 monolayer model, the ZO-1 protein distributed densely near the brush border and displayed an intact structure. While in cells under hypoxic condition, the distribution of ZO-1 was sparse. Compared with that in normoxic model, the expression level of ZO-1 in hypoxic Caco-2 monolayer was significantly reduced (Fig. 4c and d). Such down-regulated tight junction protein expression, together with the impaired brush border microvilli, greatly weakened the barrier function of Caco-2 monolayer. The TEER value measured in hypoxic model (651.6 ± 15.1 Ω cm^2^) was significantly lower than that in normoxic model (831.6 ± 26.3 Ω cm^2^).

#### 3.2.3. Enhanced absorption of SAL in hypoxic Caco-2 monolayer model

**Fig. 5:**
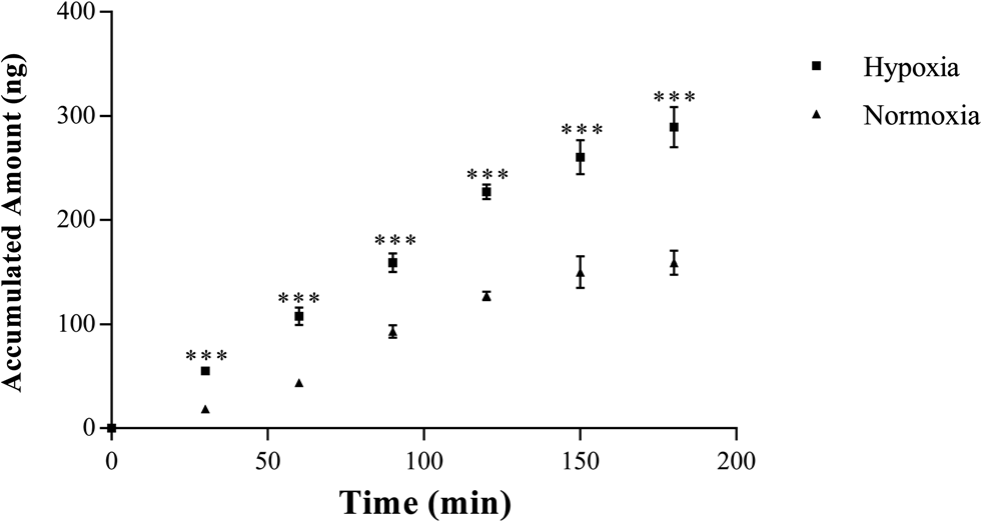
The accumulated amounts of salidroside in receiver chamber of Caco-2 cell monolayer model, *n* = 5. Salidroside was added to apical (AP) side at a final concentration of 10 μM. An aliquot of 100 μl samples were then taken from the basolateral side at 30, 60, 90, 120, 150, and 180 min after addition of salidroside. All data were reported as means ± SD. *p < 0.05, **p < 0.01, ***p < 0.001 *versus* normoxia group.

As shown in Fig. 5, the transport of SAL from apical to basolateral side in hypoxic Caco-2 monolayer model increased significantly at all measured time points when compared with that in normoxic model. The *P*_app(A→B)_ of hypoxic Caco-2 monolayer model was calculated to be (1.77 ± 0.18) × 10^−6^ cm s^−1^, which was significantly higher than that of normoxic Caco-2 monolayer model ((0.88 ± 0.11) × 10^−6^ cm s^−1^). Moreover, the *P*_app(B→A)_ of salidorside in hypoxic Caco-2 monolayer model was calculated to be (0.70 ± 0.20) × 10^−6^ cm s^−1^, which was almost 8-times higher than that of normoxic Caco-2 monolayer model ((0.88 ± 0.20) × 10^−7^ cm s^−1^). These data suggested that the permeability of SAL in hypoxic Caco-2 monolayer model was significantly increased.

**Fig. 6:**
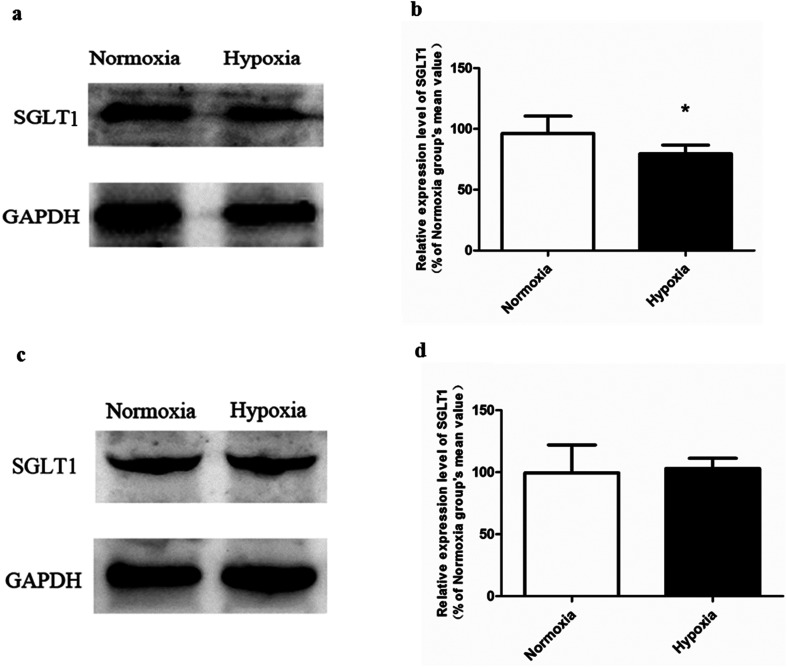
The expression of SGLT1 in Caco-2 monolayer model (a and b) and rat kidney (c and d), *n* = 5. All data were reported as means ± SD. *p < 0.05 *versus* normoxia group.

In normoxic Caco-2 monolayer model, the ratio between *P*_app(A→B)_ and *P*_app(B→A)_ was 10.0. This ratio was significantly decreased to 2.24 after addition of SGLT1 inhibitor, which suggested that SGLT1 was involved in the active transport of SAL. In hypoxic Caco-2 monolayer model, the ratio between *P*_app(A→B)_ and *P*_app(B→A)_ was 2.53, which was 4-times lower than that in normoxic condition. These data, together with our findings from western blotting (Fig. 6a), suggested that the expression of SGLT1 in hypoxic Caco-2 monolayer model was decreased.

### 3.3. Reduced hepatic metabolism of SAL in RLS derived from hypoxic rats

#### 3.3.1. CBG and SULT2A1 were involved in the deglycosylation and sulfation of SAL

**Fig. 7:**
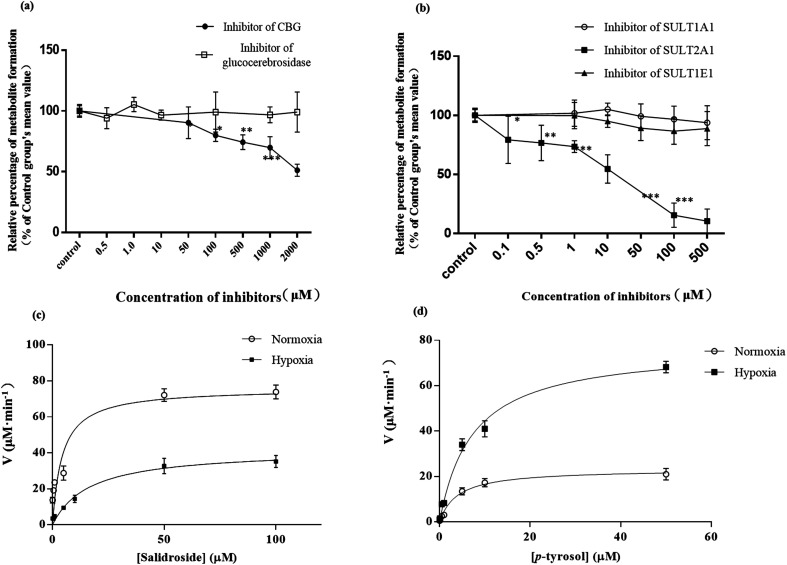
Effect of the specific β-glycosidase (a) and SULT (b) chemical inhibitors on the formation of *p*-tyrosol sulfate in RLS, *n* = 5. The value calculated from each inhibitor group was normalized by that from the control (without any inhibitor). Each data point is expressed as mean ± SD. *p < 0.05, **p < 0.01, ***p < 0.001 *versus* control. Kinetic plots for the formation of *p*-tyrosol sulfate in incubations of RLS with a serial concentrations of salidroside (c) or *p*-tyrosol (d), *n* = 5. Each data point is expressed as mean ± SD.

Since *p*-tyrosol sulfate was the most abundant metabolite of SAL *in vitro* and *in vivo*, the potential β-glycosidase and SULT isozymes responsible for the deglycosylation and sulfation of SAL in rats were identified by incubations of SAL or *p*-tyrosol with various β-glycosidase and SULT isozymes inhibitors, respectively (Fig. 7a and b).^10,11^ It was noticed that the formations of *p*-tyrosol sulfate were only dramatically inhibited when SAL was incubated with RLS in the presence of CBG inhibitor, suggesting the potential roles of CBG in the deglycosylation of SAL in rats. Moreover, the formations of *p*-tyrosol sulfate were significantly inhibited when *p*-tyrosol was incubated with RLS in the presence of SULT2A1 inhibitor, suggesting the potential roles of SULT2A1 in the sulfation of SAL in rats.

#### 3.3.2. The decreased formation of *p*-tyrosol sulfate in RLS derived from hypoxic rats

SAL was incubated in the RLS system and the formation of *p*-tyrosol sulfate was monitored to assess the hepatic metabolism of SAL. Compared with the normoxic group, the formation of *p*-tyrosol sulfate in hypoxic RLS was significantly decreased (Fig. 7c), suggesting the suppressed hepatic metabolism of SAL in hypoxic rats. Specifically, two steps including deglycosylation and sulfation were involved in the process of *p*-tyrosol sulfate formation. Among them, deglycosylation was an essential prerequisite for the next-step metabolism.^30^ Sulfation of *p*-tyrosol happened immediately after deglycosylation due to the chemical instability of *p*-tyrosol. In view of these, the data shown in Fig. 7c could be fitted into the Michaelis–Menten equation for assessing the kinetics of CBG. The *V*_max_, *K*_m_, and Cl_int_ values of CBG was calculated to be 79.9 ± 12.4 nmol min^−1^ mg^−1^, 5.2 ± 0.6 μM, and 15.4 ± 4.1 μl min^−1^ mg^−1^ in normoxic RLS, respectively. The activity of CBG in hypoxic RLS was remarkably suppressed with *V*_max_, *K*_m_, and Cl_int_ values of 47.1 ± 7.3 nmol min^−1^ mg^−1^, 19.5 ± 3.1 μM, and 2.4 ± 0.3 μl min^−1^ mg^−1^, respectively. In addition, *p*-tyrosol was used as substrate in the RLS incubation system and the formation of *p*-tyrosol sulfate was monitored to assess the kinetics of SULT2A1. Compared with that in normoxic group, the formation of *p*-tyrosol sulfate in hypoxic RLS was significantly increased (Fig. 7d). The *V*_max_ and Cl_int_ values of SULT2A1 was increased from 23.3 ± 4.4 nmol min^−1^ mg^−1^ and 5.8 ± 1.2 μl min^−1^ mg^−1^ in normoxic RLS to 78.3 ± 11.2 nmol min^−1^ mg^−1^ and 10.4 ± 2.7 μl min^−1^ mg^−1^ in hypoxic RLS, respectively.

#### 3.3.3. Expression of CBG, SULT2A1 and PXR in normoxic and hypoxic rats

**Fig. 8:**
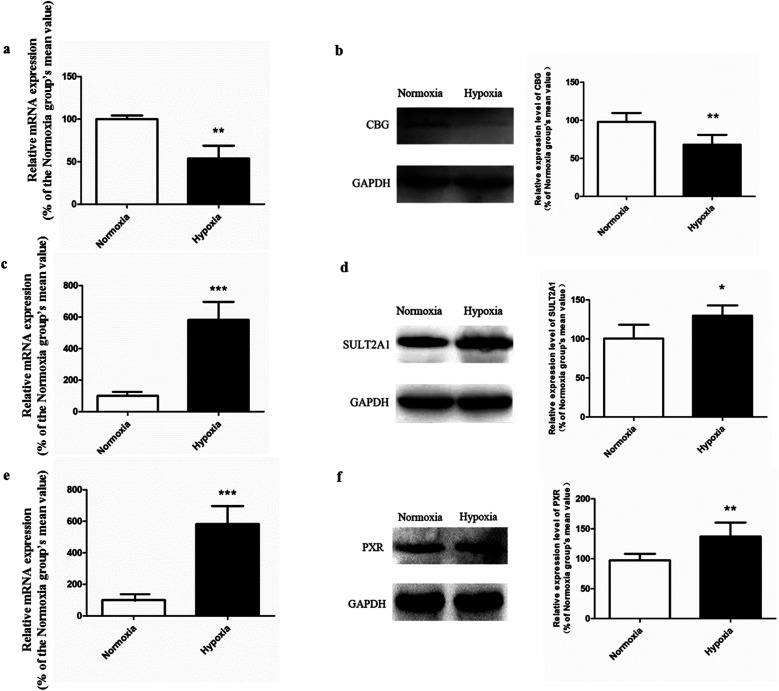
The mRNA and protein expression of CBG (a and b), SULT2A1 (c and d), and PXR (e and f) in both normoxic and hypoxic rat liver, *n* = 5. Each data point is expressed as mean ± SD. *p < 0.05, **p < 0.01, ***p < 0.001 *versus* normoxia group.

Compared with normoxic control, the mRNA and protein expression of CBG in hypoxic rat liver were significantly decreased (Fig. 8a and b). Such down-regulated expression of CBG could be used as an explanation for the reduced hydrolysis metabolism of SAL in hypoxic rat. The mRNA and protein expression of SULT2A1 in hypoxic rat liver were up-regulated compared with that in normoxic control (Fig. 8c and d), suggesting an enhanced phase II conjugation which transferring *p*-tyrosol to *p*-tyrosol sulfate in hypoxic rat. In addition, the expression of PXR, an *in vivo* master regulator of SULT2A1, was also significantly increased in hypoxic rat liver (Fig. 8e and f).

### 3.4. Reduced renal clearance of SAL in hypoxic rats

#### 3.4.1. Decreased renal blood flow resulted in reduced renal clearance of SAL in hypoxic rats

**Fig. 9:**
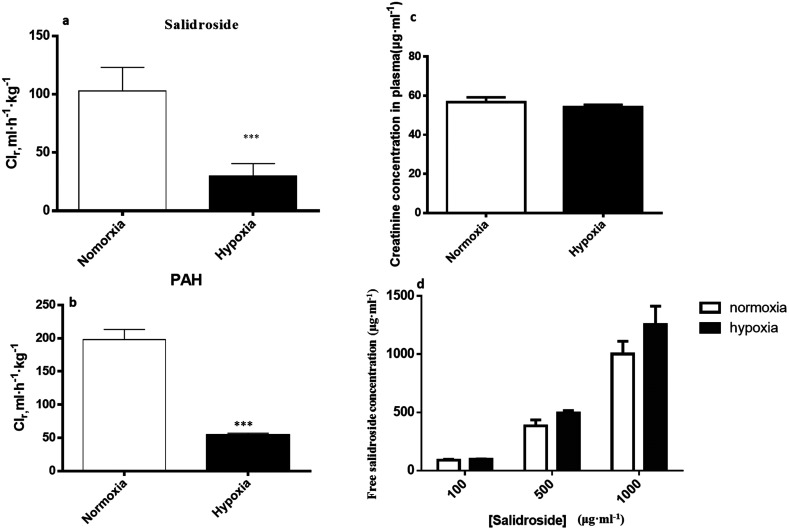
The renal clearance of salidroside (a) and PAH (b); the serum concentration of creatinine (c), and the concentration of unbound salidroside (d) in rat plasma after incubation (plasma binding) and ultrafiltration, *n* = 5. CL_r,_ renal clearance. Each data point is expressed as mean ± SD. *p < 0.05, **p < 0.01, ***p < 0.001 *versus* normoxia group.

As shown in Fig. 9a, the renal clearance of SAL in hypoxic rats (29.8 ± 7.6 ml h^−1^ kg^−1^) was significantly lower than that in normoxic rats (106.2 ± 28.5 ml h^−1^ kg^−1^). Since PAH is a non-toxic molecule that is neither bound to plasma proteins nor permeable to erythrocyte membranes, it is commonly used as a marker for assessing renal plasma flow.^14^Fig. 9b demonstrated that the renal clearance of PAH in hypoxic rats (49.1 ± 6.9 ml h^−1^ kg^−1^) was significantly lower than that in normoxic rats (196.8 ± 18.4 ml h^−1^ kg^−1^), suggesting a slower renal plasma flow in hypoxic rats when compared with that in normoxic rats. The serum concentration of creatinine in hypoxic and normorxic rats seemed to be identical (Fig. 9c), which indicated a comparable glomerular filtration rate (GFR) between hypoxic and normoxic rats. Since only unbound SAL could be metabolized and excreted, we also measured and compared the plasma binding ratio of SAL in hypoxic and normoxic rats. Fig. 9d indicated the similar plasma binding ratio of SAL in hypoxic and normoxic rats at three different spiking concentrations.

#### 3.4.2. Unchanged expression level of SGLT1 in the kidney of hypoxic rats

Our above findings suggested that SAL was the substrate of SGLT1. Since SGLT1expressed also in the kidney of rats, western blotting was performed to measure the expression levels of SGLT1 in the kidney of hypoxic and normoxic rats (Fig. 6b). It was found that the expression of SGLT1 in the kidney of hypoxic and normoxic rats was comparable.

## 4. Discussion and conclusion

The promising potential benefits of SAL in human, especially in alleviating high altitude sickness, boost investigations on its pharmacokinetics and biological activity.^2–4^ However, the absorption and disposition process of SAL under hypoxic condition has never been explored. Since accumulating studies demonstrate that hypoxia triggers a sequence of physiological changes *in vivo*, it is critical to monitor the pharmacokinetic profile of SAL in hypoxic rats with an aim for assessing its safety dose in clinical application.^1,12,13^ When prescribed for preventing high altitude sickness, the maximum oral dose of Rhodiola standard extract is about 400 mg per person per time (twice per day) and the equivalent rat dose (40 mg kg^−1^ per time) is obtained by dosage translation from human to rat according to literature guidance.^31,32^ The oral dose of SAL used in the current study (4.44 mg kg^−1^) was designed based on its content percentage in Rhodiola extract (11.1%) measured in our preliminary study. The enzyme activities of LDH, CS and the protein expression of HIF-1α were monitored for hypoxic model validation. LDH and CS are key enzymes involved in the process of glycolysis and tricarboxylic acid cycle, respectively. The increased LDH activity and decreased CS activity indicated a conversion of ATP synthesis approach from aerobic metabolism to anaerobic glycolysis. The up-regulated HIF-1α level also evidenced the successfully established hypoxic rat model and Caco-2 cell model.

Our pharmacokinetic findings indicated that after intravenous administration, the *in vivo* clearance of SAL in hypoxic rats was significantly reduced; meanwhile, the systemic exposure of SAL in hypoxic rats was more than doubled. Since the *in vivo* clearance of SAL was primarily composed by metabolic clearance and renal clearance, we further studied the hepatic metabolism and renal function of hypoxic rats.^11^*p*-Tyrosol sulfate was reported as the predominant metabolite of SAL in rats, accounting for over 50% of the total metabolites' amounts, and it was the only metabolite detected by us after RLS incubation.^11^ The formation of *p*-tyrosol sulfate was therefore used as an indicator for the hepatic metabolism of SAL. Similar with most of the phenolic glycosides, deglycosylation is an essential prerequisite for the subsequent metabolism of SAL.^30^ β-Glucosidases are thought to play roles in deglycosylation of glycolipids and dietary glucosides. There are several types of β-glucosidases existing in mammalian, including lactase-phloridzin hydrolase (LPH), cytoplasmic β-glucosidase (CBG), human acid β-glucosidase (GBA1) and bile acid β-glucosidase (GBA2). Among them, CBG was identified as a major contributor for the deglycosylation of SAL in our study. This enzyme locates most abundantly in hepatocytes, and plays an important role in detoxifying xenobiotics by hydrolyzing the l-glucoside moiety to provide a site for phase II conjugation.^33^ The *K*_m_ of CBG for SAL deglycosylation was 5.2 ± 0.6 μM, which was extremely low when compared with that for other phenolic glycosides such as quercetin 4-glycoside (65 μM) and daidzein 4-glycoside (50 μM).^34^ The lower *K*_m_ value suggests a relatively higher affinity of CBG to SAL, which could be an explanation for the rapid metabolism of SAL after administration to rats or incubated in RLS. In hypoxic RLS, the activity and expression of CBG was significantly down-regulated, leading to the decreased metabolism of SAL. Since the content of CBG in human liver is about 17.3-times higher than that in rat liver, the inhibitory effect of hypoxia on CBG would be more significant in human.^33^ We also identified SULT2A1 as one of major sulfotransferase isozymes responsible for the sulfation of *p*-tyrosol. SULT2A1 is a sulfo-conjugating phase II enzyme expressed at high level in the liver. Unlike CBG, the activity and expression of SULT2A1 was remarkably induced in hypoxic RLS. In addition, the expression of its upstream regulator, PXR, was also induced in hypoxic RLS.^35^ A previously published study demonstrates analogous findings, in which hypoxia induces the expression of CYP3A6 *via* activating its upstream regulator, constitutive androstane receptor (CAR).^36^ The activation of CAR is supposed to be correlated with the increased HIF-1α level in the plasma of hypoxic rabbits.^36^ Since CAR and PXR are all nuclear receptors mediating metabolism of xenobiotics, a potential positive correlation between the increased HIF-1α level and the up-regulated PXR expression deserves further investigation.

**Fig. 10:**
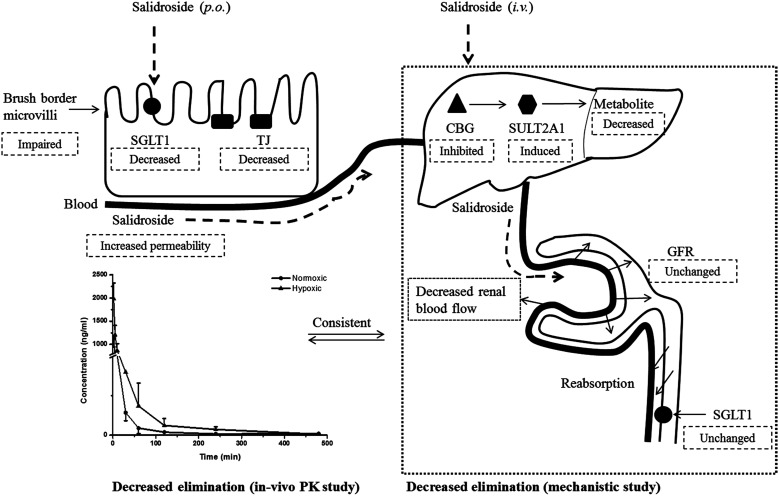
The proposed mechanism by which salidroside was absorbed and disposed in both normoxic and hypoxic rats. Although the expression of SGLT1, an up-take transporter involved in the absorption of salidroside, was decreased in hypoxic Caco-2 cell, the permeability of salidroside in hypoxic Caco-2 cell was still increased. That was because the barrier function of Caco-2 monolayer was weaken under hypoxic condition due to the impaired brush border microvilli and decreased TJ protein (ZO-1) expression. Metabolism of salidroside in rat liver was catalyzed by two enzymes, CBG and SULT2A1. Among them, deglycosylation was a crucial step for salidroside metabolism. CBG in hypoxic rat liver was inhibited, leading to the reduced metabolism of salidroside. Hypoxia had no significantly effect on the plasma binding and GFR of salidroside, while it significantly decreased the renal blood flow and therefore resulted in the decreased renal clearance of salidroside. Findings from our mechanistic study were fairly consistent with outcomes obtained from *in vivo* pharmacokinetic investigations. SGLT1, sodium-dependent glucose transporter; TJ, tight junction protein (ZO-1); CBG, cytosolic β-glucosidase; SULT2A1, sulphotransferase 2A1; GFR, glomerular filtration rate; p.o., oral administration; i.v., intravenous administration; PK, pharmacokinetics. Dashed box represents alteration in hypoxic group.

Renal excretion is a major elimination route composed by three processes, including glomerular filtration, tubular secretion and tubular re-absorption. The renal clearance (CL_r_) of SAL in normoxic rats was 106.2 ± 28.5 ml h^−1^ kg^−1^ in our study, which was not significantly higher or lower than the GFR values reported in the literature (going from 84.0 to 109.8 ml h^−1^ kg^−1^).^37^ Such finding suggests that the renal excretion of SAL in rats was primarily contributed by glomerular filtration. The CL_r_ value in hypoxic rats decreased to 29.8 ± 7.6 ml h^−1^ kg^−1^, indicating an altered renal function of rats under hypoxic condition. It is generally acknowledged that effective renal plasma flow (ERPF) and glomerular filtration rate (GFR) are the standard quantitative parameters for determining renal functions. Among various techniques and biomarkers, clearances of *p*-aminohippuric acid (PAH) and serum concentration of creatinine are considered to be the reference measurement for the assessment of ERPF and GFR, respectively.^25^ The serum creatinine in normaxic and hypoxic rats was identical in our study, indicating that the GFR of SAL in hypoxic rats had no significant change. The clearance of PAH decreased significantly in hypoxic rats, indicating the remarkably suppressed ERPF in hypoxic rats. Similar finding was observed from study on fetal lambs, in which the blood flow to renal was greatly suppressed under hypoxia condition.^38^ Furthermore, abnormal plasma protein binding ratio and changes in renal transporters may also affect renal clearance. Our findings suggested that, in normaxic and hypoxic rats, the plasma protein binding ratio and SGLT1 expression level in kidney was comparable. In view of these, we made our conclusion that the decreased CL_r_ of SAL in hypoxic rats was attributed to the suppressed ERPF. Compared with metabolic elimination, the renal clearance of SAL has been considered a minor elimination pathway accounting for no more than 20% of the eliminated SAL. However, in cases of detoxification of SAL and its metabolites, the renal clearance seems to be more important. Decreased renal clearance may expose hypoxic rats to a high risk of intoxication, the phenomenon of which was exactly observed in our preliminary trials by over dosing of SAL to rats. Our mechanistic studies demonstrated the decreased CBG activity and suppressed ERPF in hypoxic rats, which together resulted in the reduced elimination of SAL. Findings from mechanistic study were fairly consistent with our findings obtained from pharmacokinetic investigations in rats (Fig. 10).

Our pharmacokinetic findings indicated that after oral administration, the *C*_max_ and *T*_max_ values of SAL in hypoxic rats were close to those in normoxic rats. As to mechanistic investigations, SGLT1 was identified as an up-take transporter involved in the absorption of SAL with the aid of Caco-2 monolayer model. This *in vitro* finding was consistent with rat *in situ* findings reported by He *et al.*^9^ SGLT1 located primarily in the epithelial brush border microvilli and was greatly impaired under hypoxic condition due to the abnormal morphology of hypoxic Caco-2 cell.^29^ There were also SGLT1 located in kidney, the expression of which showed no significant change under hypoxia. Although the expression of SGLT1 was decreased in hypoxic Caco-2 cell, the permeability of SAL in hypoxic Caco-2 cell was still increased. That was probably due to the impaired barrier function of Caco-2 monolayer under hypoxic condition caused by the destroyed brush border microvilli and decreased TJ protein (ZO-1) expression.

In conclusion, *in vivo* pharmacokinetic investigations highlighted the enhanced systemic exposure and reduced plasma clearance of SAL in hypoxic rats. Mechanistic studies conducted subsequently gave an explanation for the distinct pharmacokinetics of SAL under hypoxic condition. Specifically, under hypoxic condition, the barrier function of Caco-2 monolayer was weakened due to the impaired brush border microvilli and decreased TJ protein (ZO-1) expression. Hepatic metabolism of SAL in hypoxic rats was attenuated because of the decreased activity and expression of CBG. Moreover, the renal clearance of SAL was reduced in hypoxic rats due to the suppressed blood flow to renal. The reduced hepatic metabolism, together with the decreased the renal clearance, concertedly contributed to the reduced plasma clearance and enhanced systemic exposure of SAL in hypoxic rats. These findings suggested the potential needs for dose-adjustment of SAL or its structural analogs under hypoxic conditions.

## Author contributions

Participated in research design: Xue, Ge and Qi. Conducted experiments: Qi, Zhao, Ge, Ma and Xu. Contributed new reagents or analytic tools: Xue and Li. Performed data analysis: Ge, Qi and Xue. Wrote or contributed to the writing of the manuscript: Ge, Qi and Xue.

## Conflicts of interest

There are no conflicts to declare.

## Abbreviations

CBG: Cytosolic β-glucosidase
CL_r_: Renal clearance
CS: Citrate synthase
ERPF: Effective renal plasma flow
GFR: Glomerular filtration rate
HIF-1α: Hypoxia inducible factor-1α
LDH: Lactate dehydrogenase
PAH: *p*-Aminohippuric acid
*P*_app_
: Apparent permeability coefficient
PAPS: 3′-Phosphoadenosine-5′-phosphosulfate
PXR: The nuclear pregnane X receptor
RLS: Liver S9 fraction
SGLT1: Sodium-dependent glucose transporter
SAL: Salidroside
SULT: Sulfotransferase
ZO-1: Zonula occludens-1

## Supplementary Material
